# Consolidation in older adults depends upon competition between resting-state networks

**DOI:** 10.3389/fnagi.2014.00344

**Published:** 2015-01-09

**Authors:** Heidi I. L. Jacobs, Kim N. H. Dillen, Okka Risius, Yasemin Göreci, Oezguer A. Onur, Gereon R. Fink, Juraj Kukolja

**Affiliations:** ^1^Cognitive Neuroscience, Research Centre Jülich, Institute of Neuroscience and Medicine (INM3)Jülich, Germany; ^2^Department of Neurology, University Hospital of CologneCologne, Germany

**Keywords:** consolidation, aging, resting-state, memory, networks, fMRI, compensation, competition

## Abstract

Memory encoding and retrieval problems are inherent to aging. To date, however, the effect of aging upon the neural correlates of forming memory traces remains poorly understood. Resting-state fMRI connectivity can be used to investigate initial consolidation. We compared within and between network connectivity differences between healthy young and older participants before encoding, after encoding and before retrieval by means of resting-state fMRI. Alterations over time in the between-network connectivity analyses correlated with retrieval performance, whereas within-network connectivity did not: a higher level of negative coupling or competition between the default mode and the executive networks during the after encoding condition was associated with increased retrieval performance in the older adults, but not in the young group. Data suggest that the effective formation of memory traces depends on an age-dependent, dynamic reorganization of the interaction between multiple, large-scale functional networks. Our findings demonstrate that a cross-network based approach can further the understanding of the neural underpinnings of aging-associated memory decline.

## Introduction

One of the most often expressed complaints of older people is having increasing difficulties with learning and remembering information. Being able to retrieve a specific piece of information from one's memory does not only involve learning of this information, but also consolidating (latin for “to make firm”) and storing it. Of all memory systems, episodic memory shows the strongest decline after the age of 60 (Ronnlund et al., 2005). This age-related episodic memory decline has been related to functional changes in distinct brain regions and networks during both encoding and retrieval (Craik and Bialystok, 2006; Daselaar et al., 2006; Miller et al., 2008; Addis et al., 2010; Craik and Rose, 2012). From these studies we know that age-related memory changes are not only associated with local deficits, but more importantly also with changes in large-scale functional brain networks (Andrews-Hanna et al., 2007). With aging, these large-scale brain networks show reduced connectivity, efficiency and changes in dynamics such as a reduction in between-network flexibility (Spreng and Schacter, 2012; Yang et al., 2013; Dennis and Thompson, 2014).

Consolidation is the progressive post-acquisition stabilization of long-term memory and the memory phases during which this presumed stabilization takes place (Dudai, 2004). In contrast to encoding and retrieval, consolidation is not associated with a conscious experience or with an explicit cognitive attribute. Therefore, consolidation is difficult to tap into and to date no cognitive paradigm exists that captures the neural correlates of consolidation directly. Traditionally, it is believed that sleep is necessary to consolidate information and to induce changes at the systems level (Euston et al., 2007; Girardeau et al., 2009). Consolidation is thought to involve a repeated spontaneous reactivation associated with the learning event during periods of sleep or rest (McGaugh, 1966). This repeated reactivation stems from the interplay of neural activities between remote brain areas that were already engaged during learning. Reactivation after encoding strengthens memory traces and thereby leads to consolidation (Pennartz et al., 2002).

The standard neurobiological view of consolidation is that the hippocampal system and the relevant neocortical areas are responsible for initial consolidation, but that with time, this information is gradually restructured and becomes integrated in the neocortex and independent of the hippocampus. The hippocampus thus plays a time-limited role. Over time, the hippocampus merely acts as an index, containing only knowledge about which cortical areas are part of that memory, which facilitates retrieval (Squire, 1992; McClelland et al., 1995). An alternative hypothesis, the multiple trace theory, suggests that the hippocampus remains continuously involved in the storage and retrieval of episodic memories (Nadel et al., 2000). In this model, the entire hippocampus-neocortical system encodes the trace in a distributed manner, but with recurrent reactivations, multiple related hippocampal-dependent traces exist. Both hypotheses assume that there is some memory reorganization happening in the brain. However, whether or not the links with the hippocampus fade away or are continuously involved differs (Nadel and Bohbot, 2001; Dudai, 2004; Nadel et al., 2012).

One-way to reveal the neural mechanisms underlying consolidation is to investigate blood-oxygenated-level-dependent (BOLD) resting-state functional magnetic resonance imaging (fMRI) connectivity differences over time (Durrant and Lewis, 2009; Vincent, 2009). Several studies have therefore examined change s in the BOLD signal after one or several days (Takashima et al., 2009; Van Kesteren et al., 2010; Vilberg and Davachi, 2013).

Importantly, several studies have investigated the formation of memory traces in response to a motor learning task (Albert et al., 2009; Daselaar et al., 2010) or visuospatial memory tasks (Peigneux et al., 2006; Takashima et al., 2009; Tambini et al., 2010; Van Kesteren et al., 2010; Groen et al., 2011; Vilberg and Davachi, 2013) by comparing the BOLD resting-state signal after and before learning in young individuals. These studies revealed that learning a motor task is associated with increased resting-state connectivity in the frontoparietal and cerebellar networks post-learning (Albert et al., 2009; Daselaar et al., 2010). Learning an episodic memory task is associated with increased hippocampal-neocortical activity after encoding (Peigneux et al., 2006; Tambini et al., 2010; Van Kesteren et al., 2010; Groen et al., 2011; Vilberg and Davachi, 2013). These studies suggest that during consolidation memories become more distributed across brain regions. However, these studies adopted a region-of-interest approach, rather than a network or whole-brain approach. After a longer interval (24 h), the connectivity between the hippocampus and neocortical areas reduces, but cortico-cortical connectivity increases, providing evidence for the standard neurobiological view of consolidation (Takashima et al., 2009). Investigation of the resting-state BOLD changes immediately after encoding revealed changes in neural activity as well as in connectivity patterns between specific memory-related brain areas, which correlated with memory performance (Takashima et al., 2009; Tambini et al., 2010; Wang et al., 2012). Importantly, these studies reveal that consolidation occurs already soon after the learned event. The timescale of these processes has thus far only been investigated either after encoding or after longer periods (i.e., 24 h or more).

In summary, to date the time course and spatial extent of these processes remain poorly understood, in particular since most of these studies adopted a region-of-interest analysis rather than a network approach. However, animal studies suggest that consolidation relies on widespread brain networks, distributed topographically in the neocortex (Freeman and Gabriel, 1999). In the current study, we aim to investigate how aging influences the orchestration of different functional networks involved in initial, short-term consolidation. Within initial consolidation we aim to compare age-related temporal changes within a short timescale, minutes instead of hours. As aging is associated with local and global deficits in functional brain networks, we expect consolidation differences involving multiple resting-state networks between younger and older participants early on. This was examined by comparing intrinsic functional connectivity patterns between groups immediately after encoding compared to before encoding. Second, as inter-network flexibility reduces with aging (Spreng and Schacter, 2012), we expected that younger individuals would show higher consolidation-induced-network dynamics than older individuals, i.e., more neocortical involvement in younger than older participants when comparing resting-state activity immediately after encoding with a subsequent time period within the consolidation phase. Finally, to understand the functional relevance of the neural changes during initial consolidation, resting-state connectivity and dynamics were correlated with behavioral retrieval performance.

## Materials and methods

### Participants

Forty healthy participants were included: 20 older adults (age between 54 and 66 years, 10 women, 10 men) and 20 young participants (age between 23 and 29 years, 10 women, 10 men). Participants were recruited as part of the RIMCAD study (Retroactive Interference during Memory Consolidation in Aging and Dementia) through advertisements. Exclusion criteria were significant medical (e.g., cancer, thyrotoxicosis), neurological (e.g., epilepsy, Parkinson's disease), or psychiatric disorders (e.g., schizophrenia, depression); current use of medication which is known to influence cerebral function (e.g., anti-depressants); pregnancy or any contraindication toward MRI-scanning (e.g., metal implants, claustrophobia). The ethics committee of the Medical Faculty of the University of Cologne approved of the study. Written informed consent was provided by all participants.

All participants underwent an extensive neuropsychological assessment before MRI scanning in order to ensure the absence of cognitive deficits. The assessment included the Mini-mental state examination (MMSE) (Folstein et al., 1975), immediate and delayed recall of the German version of the Verbal Learning and Memory test (VLMT) (Rey, 1694; Helmstaedter and Durwen, 1990), Trail Making Test (TMT) part A and B (Tombaugh, 2004), and Rey Complex Figure Test (CF Rey) (Meyer and Meyers, 1995).

### MRI data acquisition

**Figure 1:**
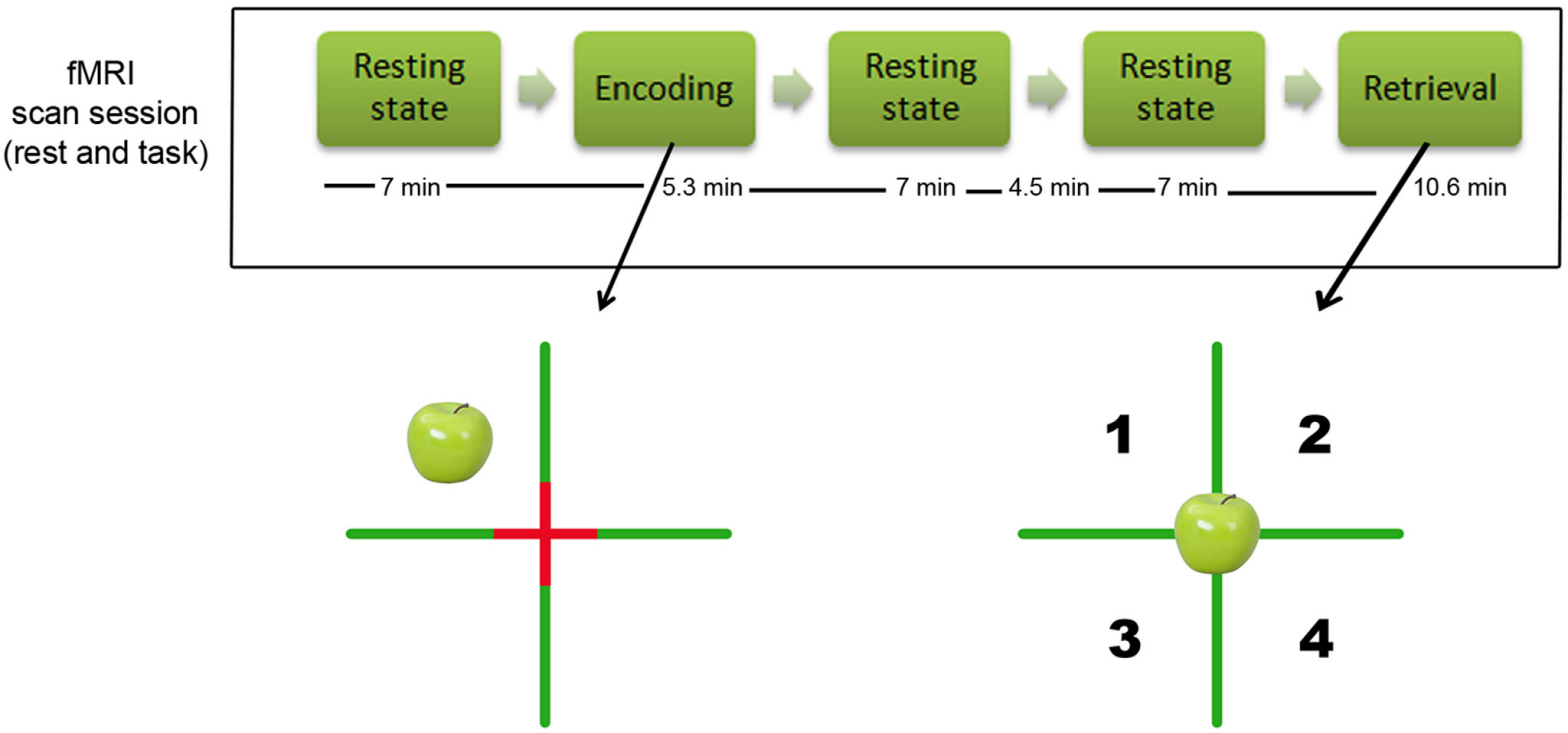
**Design of the study. Top of the figure: illustration of the design of the study and timing of the resting-state MR resting state scans**. The time indications reflect the duration of each sequence in minutes (note that there was 4.5 min pause between the two resting-state scans after encoding). **Bottom of the figure:** illustration of the episodic object-location memory task that participants performed. The left side shows the encoding condition (example of an apple in the left upper corner), the right side shows the retrieval condition in which participants had to indicate whether they had seen the object before, and if so in which quadrant.

All imaging was performed using a 3.0 Tesla Siemens Trio MRI scanner (Siemens, Erlangen, Germany) equipped with a standard head coil for radio frequency transmission and signal reception. For each participant, we acquired MR images during three resting-state sessions: one session before encoding, one immediately after encoding and one before retrieval (see Figure 1). The resting-state sessions of the after encoding and before retrieval were acquired consecutively without a pause. Each resting-state session consisted of 190 continuous T2^*^-weighted echo planar imaging (EPI) volumes (repetition time = 2200 ms; echo time = 30 ms; flip angle = 90°; 36 axial slices; matrix 64 × 64; voxel size 3.1 × 3.1 × 3.0 mm; scan time = 7 min). The second resting-state state session (after encoding) was longer (313 volumes). In order to keep the time windows comparable for the following analyses, we only used the first 190 volumes for the post encoding condition. This resulted in a time gap of 4.5 min between the after encoding resting-state session and the before retrieval resting-state session (see Figure 1). During the resting-state scan, participants were instructed to keep their eyes closed, to think of nothing in particular, and not to fall asleep. For the task-related fMRI, we applied T2^*^-weighted echo planar images (EPI) with BOLD contrast, echo time (TE) = 30 ms, repetition time (TR) = 2200 ms, flip angle = 90°, slice thickness 3.0 mm, interslice gap 0.3 mm, field of view (FoV) = 200 mm, matrix size 64 × 64, inplane resolution = 3.125 × 3.125 mm. Thirty axial slices per volume covering the brain from vertex to cerebellum were acquired sequentially.

In addition, a high-resolution T1 anatomical image (1 mm isotropic) was obtained for each subject using a three-dimensional magnetization-prepared, rapid acquisition gradient echo sequence. The T1-weighted images were used to aid the registration of the functional data into a common standard brain coordinate system (MNI152) and to account for putative atrophy differences between the groups.

### Experimental paradigm

We used an adaptation of the spatial Source Memory Task originally employed by Cansino et al. (2002) and previously employed by us (Kukolja et al., 2009). The experiment consisted of an encoding and a retrieval session (see Figure 1). Stimuli consisted of color photographs of common objects. For each subject, a different set of 64 images from a pool of 96 pictures was selected for encoding. Half of the pictures showed artificial (i.e., man-made) objects while the other half presented natural (i.e., not man-made) objects. In the retrieval session, all 96 pictures were presented. This number was used because pilot testing had shown that a large proportion of older participants had difficulties performing the task with more than 100 pictures. During the fMRI encoding session, participants saw a screen which was divided into four quadrants by a green cross with a red fixation cross superimposed at the intersection of the green lines. The 64 objects appeared successively in random order in each quadrant with equal probability (Figure 1). The pictures were shown for 2000 ms, followed by an inter-stimulus interval of 1570 ms in an event-related design. Participants were asked to memorize all objects and the quadrant in which each object appeared. To ensure proper recognition and visual processing of each object, participants had to indicate with the index or middle finger of their right hand whether the object was “natural,” e.g., an animal or a vegetable, or “artificial,” e.g., a tool or an instrument. Thirty-two null events which merely showed the green cross with the red fixation cross superimposed at the intersection were interspersed. This effectively resulted in a variable stimulus-onset asynchrony and allowed a comparison of the BOLD signal of each event type with a no-stimulus baseline (i.e., the null events). The green cross subtended a visual angle of 63.7° horizontally and vertically. The center of the pictures was located at a visual angle of ±11° from the horizontal and vertical midline, respectively. The pictures themselves subtended visual angles from 7.3° to 22.0° horizontally and from 11.4° to 19.9° vertically.

During the fMRI retrieval session, all 96 pictures were presented randomly at the center of the screen (Figure 1). Thus, in addition to the 64 objects previously shown during the encoding session, 32 new objects (16 natural, 16 artificial) were interspersed. The participants' task was to decide whether each picture presented had been shown during the encoding session (“old”) or not (“new”). Participants were asked to respond with their left index finger if the picture was new. If the picture was judged as old, participants were asked to indicate the quadrant in which the object had been presented by pressing one of four keys using their right index to little finger. The quadrants were numbered from 1 to 4 (1 corresponding to the index finger and 4 corresponding to the little finger) to facilitate response selection. Participants were instructed to make a guess regarding the object's position if they were not sure about the position which the object had occupied during encoding. For subsequent analyses of accuracy and reaction times during retrieval, only items followed by a correct response in the encoding session (i.e., whether the item was artificial or natural) were taken into account. In the retrieval session, the baseline picture also consisted of the green cross with the red fixation cross superimposed at its intersection, dividing the screen into four quadrants. Similar to the encoding session, 48 null-events were interspersed randomly. Pictures were presented for 1500 ms, followed by a 2650 ms inter-stimulus interval in an event-related design. The durations of the encoding and retrieval sessions were 5.3 and 10.6 min, respectively.

Stimuli were shown using Presentation software (Neurobehavioral Systems Inc., San Francisco, CA, USA). Prior to the fMRI experiment, participants performed one training session of the encoding and retrieval tasks outside and another one inside the MR scanner, both with a reduced number of items.

Age-related activation differences during performance of this task have been described in prior work (Kukolja et al., 2009). For the current study, we focused on brain network connectivity during consolidation and its influence on behavioral performance. For reasons of completeness, we have additionally analyzed brain activity during encoding and retrieval parts of the episodic memory task. The methods and results are provided in the Supplemental Data (see Supplemental Methods, Supplemental Data, Supplemental Tables 1, 2 and Supplemental Figures 1–3).

### Data analysis

#### Analysis of behavioral data

Behavioral data were analyzed with the SPSS version 20.0. Demographic and cognitive group differences were investigated with ANOVA for continuous variables and a *X*^2^ test for categorical variables. For the functional task, we applied a signal detection analysis to investigate aging related differences in “old” vs. “new” judgments. This analysis determines the sensitivity *d*′ representing the ability to classify old items as “old” (Green and Swets, 1966). This distinction cannot be assessed by the comparison of response accuracy. The *d*′ index computes the distance between the signal (old as classified as old) and noise (old classified as new) distribution means in standard deviation units. The parameter was calculated as described in (Stanislaw and Todorov, 1999; Kukolja et al., 2009): *d*′ = Φ^−1^(*H*′)− Φ^−1^(*F*′) where *H*′ is the corrected hit rate, *F*′ the corrected false alarm rate, and Φ − 1 is the function converting probabilities into *z* scores. To protect against ceiling effects with *H* of 1 and *F* of 0 (corresponding *z*-values would be +∞ or −∞, respectively), we used corrected values of *H* and *F*: *H*′ = (*h* + 0.5)/(*h* + *m* + 1) and *F*′ = (*f* + 0.5)/(*f* + *cr* + 1) where *h* is the number of hits (old as “old”), *m* the number of misses (old as “new”), *f* the number of false alarms (new as “old”), and *cr* is the number of correct rejections (new as “new”).

To investigate the functional relevance of the resting-state findings (third hypothesis), partial correlations, corrected for demeaned gray matter volume, between functional connectivity and the experimental task performance (*d*′ prime) were calculated. Statistical significance threshold was set at *p* < 0.05.

#### Data preprocessing

Data analysis was carried out using FMRIB's Software Library (FSL version 5.0.4; www.fmrib.ox.ac.uk/fsl). Preprocessing consisted of motion correction (MCFLIRT) (Jenkinson et al., 2002), removal of non-brain tissue (using the Brain Extraction Tool) (Smith, 2002), spatial smoothing using a 5 mm full-width-at-half-maximum Gaussian kernel, and high-pass temporal filtering equivalent to 100 s (0.01 Hz). After preprocessing the fMRI volumes were registered to the subject's high-resolution T1-weighted scan using affine registration (FLIRT) with 6 degrees of freedom. Subsequent registration was performed to standard space (MNI152) images using an initial FLIRT affine registration and then further refined with non-linear registration (FNIRT) with a warp resolution of 10 mm and 12 degrees of freedom. Data were resampled to 3 mm^3^ resolution.

An overview of all the processing steps of the resting-state data is depicted in Supplemental Figure 4.

#### Independent component analyses (ICA)

The concatenated pre-processed data were analyzed using the Multivariate Exploratory Linear Optimized Decomposition into Independent Components (MELODIC) (Beckmann and Smith, 2004) employing the multi-session temporal concatenation ICA to identify large-scale patterns of functional connectivity across participants. This method automatically estimates and decomposes the data from all 120 scans (both groups at three time points) into a set of spatially independent maps each associated with an internally consistent temporal dynamic characterized time course. These spatio-temporal patterns reflect the underlying resting-state networks and artifactual components that give rise to the BOLD signals. The ICA provides intensity values (*z*-scores) and thus a measure of the contribution of the time course of a component to the signal in a given voxel. This allows a voxel-wise map of quantitative measures of functional connectivity that can be further statistically analyzed with the dual regression technique. ICA is superior to seed-based correlations for probing large-scale resting-state networks as it is more robust to cardiovascular confounds or motion artifacts (Beckmann and Smith, 2004). The fact that we used the data of all subjects and all time points ensures a robust identification of the components.

We focused on networks relevant to memory or consolidation, viz. the default mode network, the frontoparietal network, the executive network and the salience network. Furthermore, as we used a visuospatial memory task with objects, we also included the ventral stream network and two visual networks. The components of interest were selected based on visual inspection and spatial correlation with a previously defined template of 10 well-matched resting-state components to the BrainMap database (Smith et al., 2009; Laird et al., 2011). To account for noise, components reflecting the white matter and cerebrospinal fluid were included in further analyses (Cole et al., 2010; Birn, 2012). Statistical analyses involving all datasets were carried out using the dual regression procedure.

#### Dual regression

To investigate functional connectivity across participants and time points, the individual subject-specific spatial maps and the time courses corresponding to the chosen 10 independent components (IC) (eight components of interest and two components to account for noise) were voxel-wise estimated using the dual regression procedure (Beckmann et al., 2009; Filippini et al., 2009). First, for each subject, the group-average set of spatial maps was regressed (as spatial regressors in a multiple regression) into the subject's 4D space-time dataset. This resulted in a set of subject-specific time series, one per group-level spatial map (stage 1). Next, those time series were regressed (as temporal regressors, again in a multiple regression) into the same 4D dataset, resulting in a set of subject-specific spatial maps, one per overall group-level spatial map (stage 2). In order to analyze differences between resting-state connectivity before encoding and after encoding, and after encoding and before retrieval, we subtracted individual spatial maps from the stage 2 dual regression. In particular, we created subject-specific maps for “after encoding minus before encoding” and for “before retrieval minus after encoding.” First, we analyzed within (young or old) and between (young vs. old) group differences in resting-state connectivity in the 8 components for the time point before encoding, as a reference and to validate our results with the existing literature. For the first hypothesis, we then tested within group (young or old) and between group differences (young vs. old) in resting-state connectivity for the 8 components of interest for the “after encoding minus before encoding” maps with two-sample unpaired *T*-tests. For the second hypothesis, we performed similar analyses, but then for the “before retrieval minus after encoding” maps. As we used the maps of the subtracted time points, these group comparisons reflect the interaction “group by time” (Nieuwenhuis et al., 2011). In all these analyses, gray matter density (see below) was added as a centered covariate. For these analyses, we included all voxels in the brain (whole brain connectivity patterns). To assess within-network functional connectivity differences, we performed the same analyses, but with the spatial maps of the IC as a spatial boundary in the multiple regressions. Non-parametric permutation tests (5000 permutations; FSL randomize) were used to detect statistically significant differences within or between the groups (Nichols and Holmes, 2002; Smith et al., 2004). Finally, a family-wise error (FWE) correction for multiple comparisons was performed, implementing threshold-free cluster enhancement (TFCE) (Smith and Nichols, 2009) using a significance threshold of *p* < 0.05 (Smith and Nichols, 2009). The regions that showed differences in functional connectivity between groups were visualized by means of FSLview. *z*-Values were extracted from significant clusters of voxels from each individual spatial map (FWE-corrected *p* < 0.05). These values represent connectivity to the given resting-state network with higher absolute *z*-values reflecting stronger connectivity to the resting-state network. Individual resting-state connectivity values for the clusters of each component were exported to and correlated in SPSS (see Section Analysis of Behavioral Data) with behavioral performance to answer hypothesis 3. Anatomical labeling was done with the Harvard-Oxford atlas and the Cerebellum atlas available in FSL.

#### Voxelwise-based gray matter volume correction

To statistically account for possible effects that could be explained by local structural differences between older and young participants, we added partial volume information of gray matter volume at each voxel as a subject-wise and voxelwise regressor in the dual regression analyses (Oakes et al., 2007). To that end, a four-dimensional (4D) map was created by concatenating every participant (4D) with the feat_gm_prepare script of FSL. The purpose of this method is to isolate the functional changes component which cannot be attributed to anatomical differences and is thus likely due to genuine functional differences.

#### Between-network connectivity

Between-network functional connectivity differences were assessed using the FSLNets toolbox (http://fsl.fmrib.ox.ac.uk/fsl/fslwiki/FSLNets). The following steps were performed. First the respective time courses of all maps in each subject were extracted from the dual regression procedure and normalized by the subjects standard deviations. Time courses of artifactual components (e.g., noise) were regressed out of the individual data. Then, subject-wise correlation matrices of the full and partial correlation of all the remaining resting-state time courses were created. These correlation coefficients were then Fisher z-transformed and corrected for temporal autocorrelations. This yields individual correlation matrices of all components, representing neuronal signal corrected for the influences of artifactual components. Partial correlations provide a better approximation for direct connections, while full correlations are more sensitive to both direct and indirect connections (Marrelec et al., 2006; Smith et al., 2011). This is because the partial correlation regresses out the time course of a third region when estimating the correlation between the time courses of two other regions (Smith et al., 2013). Therefore, the partial Fisher z-transformed correlations between networks were extracted for each individual and for the three scanned time points. These were entered into the Statistical Package for the Social Sciences (SPSS Inc., Chicago, IL) version 20.0 for repeated measure ANOVA analyses. The within-group analyses were performed with time as the dependent factor (with three levels). Group-differences were calculated with time as the dependent factor with three levels (before encoding, after encoding and before retrieval) and group as independent factor. Main effect of time and group and the interaction “time by group” were analyzed. This interaction tested a differential change for group differences in network coupling. The assumption of sphericity was checked with the Mauchly test, and, if necessary, a Huynh-Feldt correction was applied. Multiple comparisons were adjusted by applying a Bonferonni correction (FWE-based). *Post-hoc* repeated contrasts and simple main effects were performed in order to explore the interaction effects.

Individual partial correlation values for the interaction between components were correlated (see Section Analysis of Behavioral Data) with behavioral performance to answer hypothesis 3. Significance threshold was set at *p* < 0.05.

## Results

### Demographics and cognitive performance

Subject characteristics are shown in Table 1. As to be expected, mean age of the older group differed significantly from that of the young group (*p* < 0.001). Differences in cognitive performance between older and young participants were found for the delayed recognition trial of the VLMT memory test (*p* < 0.01) and the delayed recall of the Rey-Osterrieth Complex Figure Test (*p* < 0.05). Older individuals had a lower *d*′ prime (*p* < 0.01) score relative to the young individuals.

**Table 1:** **Demographic and cognitive characteristics of the participants**.

	**Older (*n* = 20)**	**Young (*n* = 20)**
Age (in years)	59.3 (6.0)^***^	24.6 (2.8)
Education (in years)	16.9 (3.9)	17.7 (2.5)
Female (in %)	50	50
MMSE score	29.4 (0.7)	29.6 (0.9)
VLMT learning	51.1 (9.1)	56.4 (8.8)
VLMT memory		
delayed recall (number of words)	10.8 (2.8)	11.6 (3.0)
delayed recognition (number of words)	11.9 (3.1)^**^	13.9 (1.3)
TMT-A (s)	32.3 (8.6)	23.1 (7.1)
TMT-B (s)	64.2 (17.9)	44.1 (12.6)
CF Rey score IR	35.7 (1.2)	35.5 (0.8)
CF Rey score DR	22.1 (6.3)^*^	26.5 (4.0)
Experimental memory task: *d*′ prime	2.41 (0.70)^**^	3.01 (0.52)

Values are mean (SD). MMSE, Mini-Mental state examination; VLMT, verbal learning and memory test; TMT, trail making test; CF Rey, Rey Complex Figure Test. Differences between both age groups significant at

*** *p < 0.001*,

** p < 0.01 and

* *p < 0.05*.

### Identification of the components

**Figure 2:**
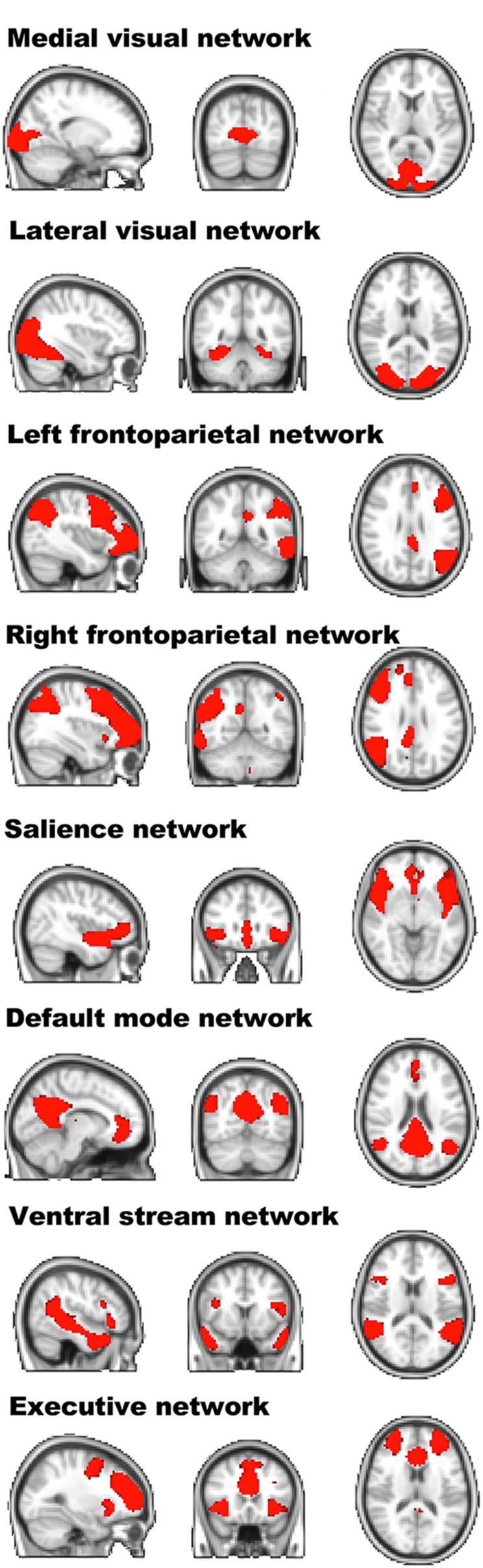
**Spatial layout of the independent components of interest of the entire sample**. Visualization of the resting-state independent components extracted from the entire sample. For each component, the sagittal, coronal, and transversal perspectives are depicted (radiological convention).

Spatial maps of the components of interest, based on the entire sample, including young and older participants, can be seen in Figure 2. Spatial correlations between our IC components and the Brain Map database (Smith et al., 2009; Laird et al., 2011) were high for the frontoparietal network (right: *r* = 0.679; left: *r* = 0.688), the default mode network (*r* = 0.834), the visual network (medial: *r* = 0.667; lateral: *r* = 0.720), and for the executive network (*r* = 0.616). The ventral and the salience networks were not represented in the 10 well-matched networks and were therefore identified via visual inspection.

### Whole brain functional connectivity differences

We first set out to investigate resting-state connectivity between these components and the whole brain during baseline (before encoding) and to compare it with post-encoding consolidation within each group. All reported effects were significant at a voxel-level family-wise error (FWE) adjusted threshold of *p* < 0.05, corrected for multiple comparisons and are reported in Montreal Neurological Institute (MNI) coordinates.

**Figure 3:**
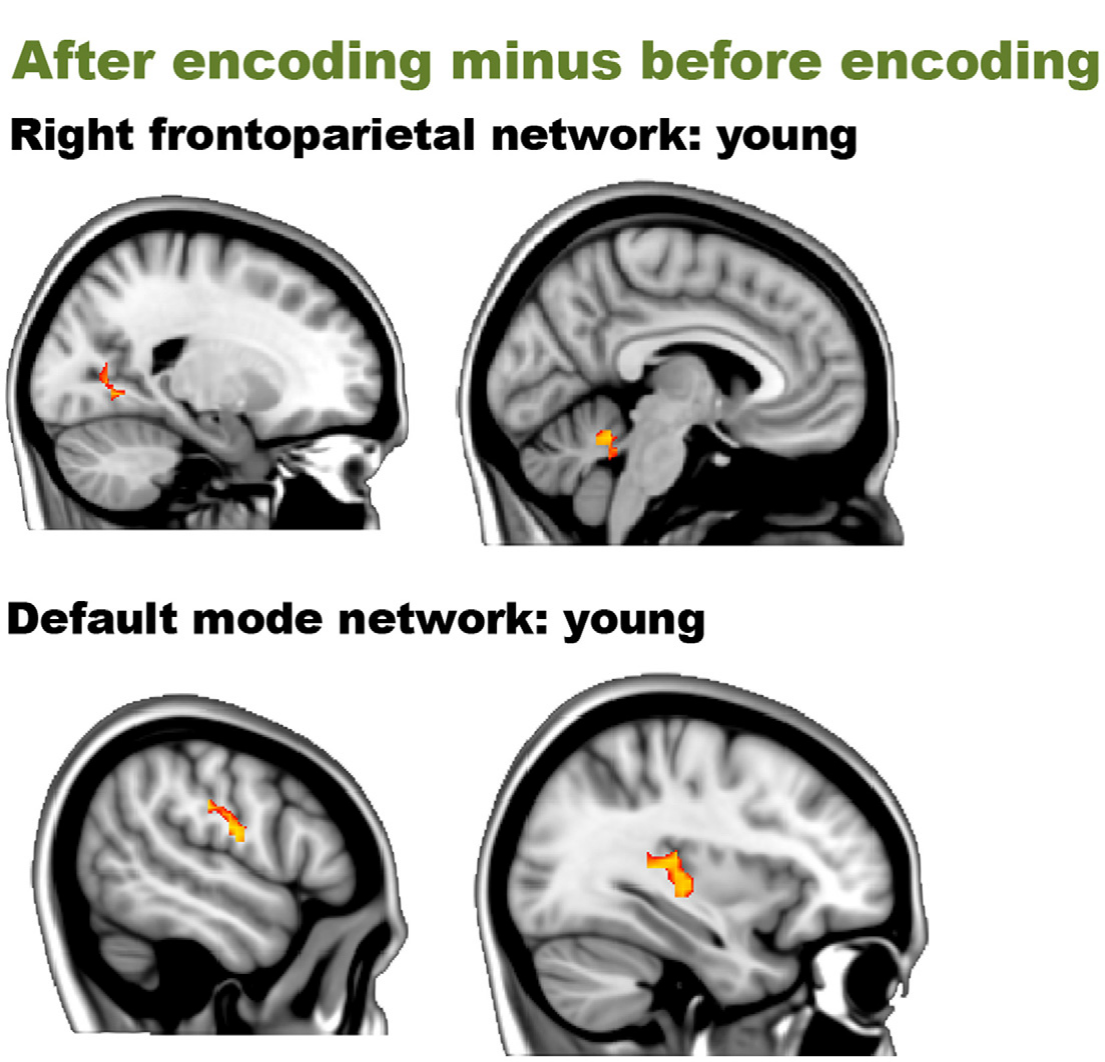
**Resting-state differences over time within young participations for the whole brain**. Whole-brain within-group effects were found for the contrast “after encoding minus before encoding” in young individuals. Red clusters indicate increased connectivity over time.

Before encoding, the young group and the old group showed widespread high levels of within-group connectivity for all the networks (all *p*s < 0.001, see Supplemental Figure 5 and Table 2). For the “after minus before encoding” condition, the young participants showed increased connectivity within the right frontoparietal network (*p* = 0.009) and the default mode network (*p* = 0.005). No significant effects were found within the old group. For the “before retrieval minus after encoding” condition, young and old participants showed no within-group effects (see Figure 3 and Table 2).

**Table 2:** **Resting-state connectivity between networks of interests and the whole brain within the young and older individuals**.

**Brain regions within the clusters**	**Hemisphere**	**Cluster size (mm^3^)**	**Peak *p*-value**	**MNI coordinates (*x, y, z*)**
YOUNG: BEFORE ENCODING^*^				
The right frontoparietal network	LH/RH	–	<0.001	–
The salience network	LH/RH	–	<0.001	–
The lateral visual network	LH/RH	–	<0.001	–
The medial visual network	LH/RH	–	<0.001	–
The ventral network	LH/RH	–	<0.001	–
The default mode network	LH/RH	–	<0.001	–
The executive network	LH/RH	–	<0.001	–
The left frontoparietal network	LH/RH	–	<0.001	–
OLD: BEFORE ENCODING^*^				
The right frontoparietal network	LH/RH	–	<0.001	–
The salience network	LH/RH	–	<0.001	–
The lateral visual network	LH/RH	–	<0.001	–
The medial visual network	LH/RH	–	<0.001	–
The ventral network	LH/RH	–	<0.001	–
The default mode network	LH/RH	–	<0.001	–
The executive network	LH/RH	–	<0.001	–
The left frontoparietal network	LH/RH	–	<0.001	–
**YOUNG: AFTER ENCODING MINUS BEFORE ENCODING**				
***The right frontoparietal network***				
Cerebellar lobule I–IV	LH	693	0.009	−3, −51, −21
Intracalcarine gyrus, precuneus	RH	82	0.024	18, −66, 3
Lateral occipital cortex	RH	78	0.031	42, −84, 24
Lingual gyrus, occipital fusiform gyrus	RH	55	0.019	21, −63, −3
***The default mode network***				
Postcentral gyrus	LH	1809	0.006	−48, −21, 30
Insular cortex, Heschl's gyrus	LH	1050	0.005	−30, −24, 0
Precentral gyrus, inferior frontal gyrus	LH	95	0.027	−48, 0, 27
Postcentral gyrus, supramarginal gyrus	RH	17	0.025	45, −15, 27
Postcentral gyrus. precentral gyrus	RH	12	0.042	51, −12, 30
Postcentral gyrus, central opercular cortex	RH	12	0.034	57, −9, 18

*p-Values refer to the peak of all significant voxels. All p-values < 0.05 are family-wise error (FWE)-corrected at 0.05. Clusters with less than 10 mm^3^ are not reported. MNI, Montreal Neurological Institute; LH, left hemisphere; RH, right hemisphere*.

* *, For the before encoding time, we have not split up the results in clusters, as the effects involved large areas of the total brain and these effects are not the main interest of the manuscript (see Supplemental Figure 5). The Harvard-Oxford (sub)cortical and cerebellum atlas provided within FSL were used as anatomical reference*.

**Figure 4:**
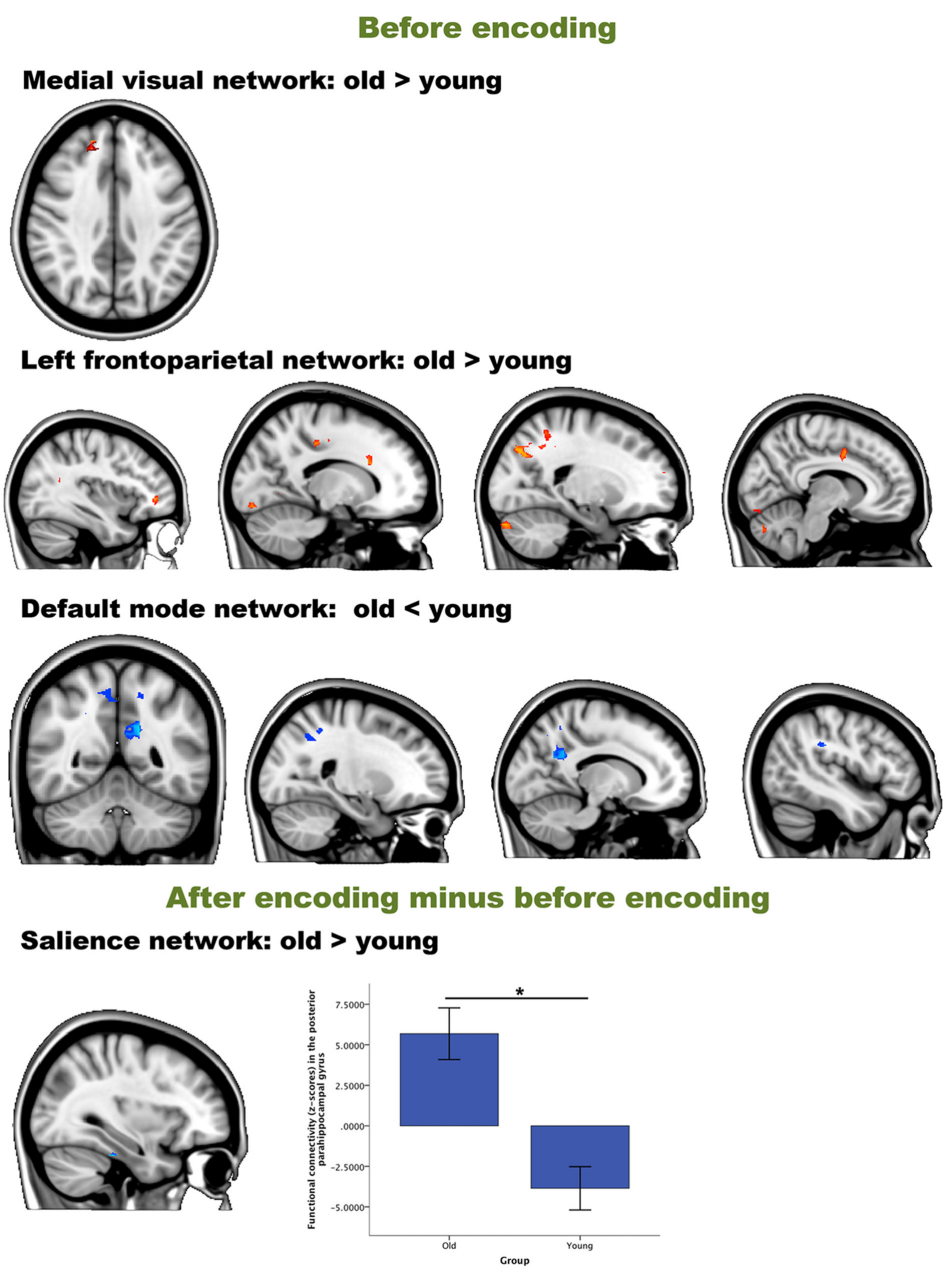
**Resting-state differences between older and young participants for the whole brain**. Whole-brain group differences were found for the contrasts “before encoding” and “after encoding minus before encoding.” Blue clusters show decreased connectivity in older participants, red clusters indicate increased connectivity in older participants. The bar chart depicts the group differences in connectivity between the parahippocampal gyrus and the executive network (error bars indicate 1 standard error). ^*^p < 0.05.

We then performed an interaction analysis “group by time point” for the three different time comparisons (see Figure 4). Before encoding, older participants showed higher connectivity between the frontal pole [peak (*x, y, z* = 15, 48, 33), *p* = 0.025] and the medial visual network compared to the young group. Furthermore, older participants also showed higher connectivity than younger participants between the left frontoparietal network and regions widespread over the cerebrum and one region in the cerebellum. Largest clusters for this increased connectivity (old > young) were observed in the lateral occipital cortex [peak (*x, y, z* = 21, −75, 39), *p* = 0.013], the crus I and II of the cerebellum [peak (*x, y, z* = 15, −84, −30), *p* = 0.015], the anterior cingulate cortex [peak (*x, y, z* = 18, 0, 39), *p* = 0.019], fusiform gyrus [peak (*x, y, z* = −12, −87, −15), *p* = 0.019], frontal pole [peak (*x, y, z* = −36, 42, 0), *p* = 0.017] and precuneus [peak (*x, y, z* = −24, −66, 12), *p* = 0.034]. Before encoding, older participants showed lower connectivity than younger participants between the default mode network and lateral occipital [peak (*x, y, z* = 30, −57, 33), *p* = 0.028], lateral parietal [peak (*x, y, z* = −45, −33, 30), *p* = 0.029], and medial parietal regions [peak (*x, y, z* = −15, −51, 27), *p* = 0.006 and peak (*x, y, z* = 6, −48, 48), *p* = 0.024] (see Table 3 and Figure 3). It is important to note that these connectivity patterns replicate previously reported age-related differences in resting-state functional connectivity (Damoiseaux et al., 2008; Sambataro et al., 2012).

**Table 3:** **Group differences in resting-state connectivity of the networks of interest with the whole brain**.

**Brain regions within the clusters**	**Hemisphere**	**Cluster size (mm^3^)**	**Peak *p*-value**	**MNI coordinates (*x, y, z*)**
**BEFORE ENCODING**				
***The medial visual network (old > young)***				
Frontal pole, superior frontal gyrus	RH	72	0.025	15, 48, 33
***The default mode network (old < young)***				
Posterior cingulate cortex, precuneus	LH	820	0.006	−15, −51, 27
Precuneus, posterior cingulate cortex, superior parietal lobule	RH	221	0.024	6, −48, 48
	LH	90	0.029	−15, −54, 51
Lateral occipital cortex, angular gyrus	RH	141	0.028	30, −57, 33
Parietal operculum, supramarginal gyrus	LH	130	0.029	−45, −33, 30
Lingual gyrus, occipital pole	LH	122	0.017	−3, −90, −18
Angular gyrus, superior parietal lobule, precuneus, lateral occipital cortex	LH	38	0.035	−18, −60, −39
***The left frontoparietal network (old > young)***				
Lateral occipital cortex, precuneus	RH	1190	0.013	21, −75, 39
Right crus I and II	RH	480	0.015	15, −84, −30
Anterior cingulate cortex,	RH	270	0.019	18, 0, 39
supplementary motor area,	LH	76	0.023	−15, 21, 24
paracingulate cortex	LH	15	0.034	−9, 36, 0
Occipital fusiform gyrus, lingual gyrus, occipital pole	LH	227	0.019	−12, −87, −15
Frontal pole	LH	110	0.017	−36, 42, 0
	LH	78	0.027	−27, 54, −3
Precuneus, intracalcarine cortex	LH	100	0.034	−24, −66, 12
Intracalcarine cortex, occipital fusiform gyrus, lingual gyrus	LH	95	0.029	−24, −75, 6
Posterior cingulate cortex, precentral gyrus, postcentral gyrus, precuneus	LH	82	0.024	−15, −30, 42
Middle frontal gyrus	RH	11	0.041	27, 18, 39
**AFTER ENCODING MINUS BEFORE ENCODING**				
***The salience network (old after encoding – old before encoding > young after encoding – young before encoding)***				
Posterior parahippocampal gyrus, posterior fusiform gyrus	RH	15	0.019	33, −30, −27

*p-Values refer to the peak of all significant voxels. All p-values < 0.05 are family-wise error (FWE)-corrected at 0.05. Clusters with less than 10 mm^3^ are not reported. MNI, Montreal Neurological Institute; LH, left hemisphere; RH, right hemisphere. The Harvard-Oxford (sub)cortical and cerebellum atlas provided within FSL were used as anatomical reference*.

In order to answer hypothesis 1, we examined whether there are age differences in immediate consolidation (after encoding) by focusing on the contrast “after encoding minus before encoding”. The dual regression with a FWE correction for multiple comparisons revealed a significant group difference for the salience network with posterior part of the parahippocampal gyrus [peak (*x, y, z* = 33, −30, −27), *p* = 0.019] (see Figure 3).

For the second hypothesis, we investigated the “before retrieval minus after encoding” contrast, and found no significant group differences for any component of interest.

To investigate the functional relevance of the resting-state connectivity group differences found during initial consolidation, we performed correlations between the individually extracted connectivity values and retrieval performance (hypothesis 3). Neither in the young, nor in the older group, did we find any significant correlation between the connectivity findings and the performance on the experimental memory task (all *p* > 0.05).

### Within network connectivity differences

To investigate the robustness of the components involved in consolidation, we examined resting-state connectivity of the same comparisons within the components, FWE-corrected for multiple comparisons.

**Figure 5:**
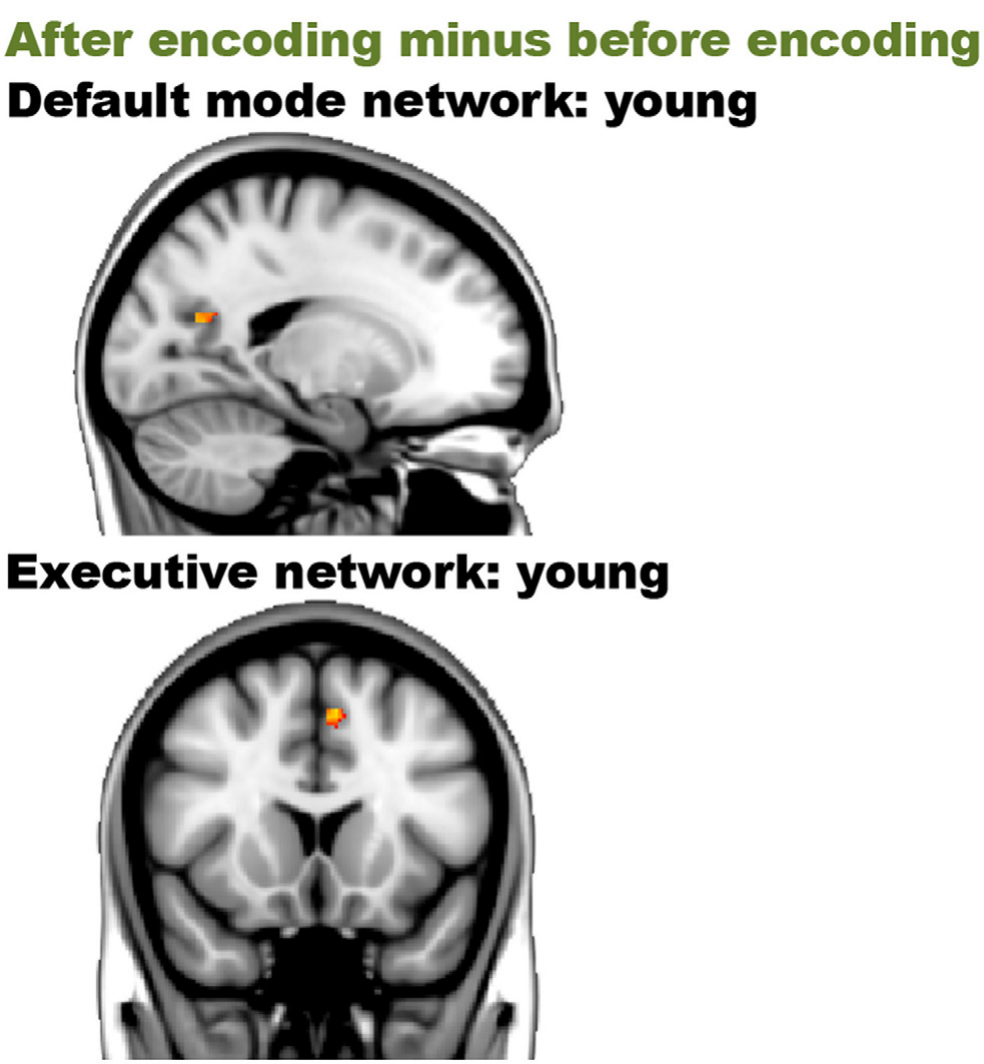
**Resting-state differences over time within young participations within the networks of interest**. Within-network within-group effects were found for the contrast “after encoding minus before encoding” in young individuals. Red clusters indicate increased connectivity over time.

Before encoding, the young group and the old group showed widespread high levels of within-group connectivity for all the networks (all *p*s < 0.001; see Supplemental Figure 6 and Table 4). For the “after minus before encoding” condition, the young participants showed increased connectivity within the default mode network (*p* = 0.02) and the executive network (*p* < 0.001). No significant effects were found within the old group. For the “before retrieval minus after encoding” condition, no effects were found within the young or within the old group (see Figure 5 and Table 4).

**Table 4:** **Resting-state connectivity within the networks of interests within the young and older individuals**.

**Brain regions within the clusters**	**Hemisphere**	**Cluster size (mm^3^)**	**Peak *p*-value**	**MNI coordinates (*x, y, z*)**
YOUNG: BEFORE ENCODING^*^				
The right frontoparietal network	LH/RH	–	<0.001	–
The salience network	LH/RH	–	<0.001	–
The lateral visual network	LH/RH	–	<0.001	–
The medial visual network	LH/RH	–	<0.001	–
The ventral network	LH/RH	–	<0.001	–
The default mode network	LH/RH	–	0.006	–
The executive network	LH/RH	–	<0.001	–
The left frontoparietal network	LH/RH	–	<0.001	–
OLD: BEFORE ENCODING^*^				
The right frontoparietal network	LH/RH	–	<0.001	–
The salience network	LH/RH	–	<0.001	–
The lateral visual network	LH/RH	–	<0.001	–
The medial visual network	LH/RH	–	<0.001	–
The ventral network	LH/RH	–	<0.001	–
The default mode network	LH/RH	–	0.011	–
The executive network	LH/RH	–	<0.001	–
The left frontoparietal network	LH/RH	–	<0.001	–
**YOUNG: AFTER ENCODING MINUS BEFORE ENCODING**				
***The default mode network***				
Precuneus	LH	199	0.017	−18, −63, 18
***The executive network***				
Paracingulate cortex, superior frontal cortex	LH	42	0.008	−6, 15, 51

*p-Values refer to the peak of all significant voxels. All p-values < 0.05 are family-wise error (FWE)-corrected at 0.05. Clusters with less than 10 mm^3^ are not reported. MNI, Montreal Neurological Institute; LH, left hemisphere; RH, right hemisphere*.

* *For the before encoding time, we have not split up the results in clusters, as the effects involved large areas of the total brain and these effects are not the main interest of the manuscript (see Supplemental Figure 6). The Harvard-Oxford (sub)cortical and cerebellum atlas provided within FSL were used as anatomical reference*.

**Figure 6:**
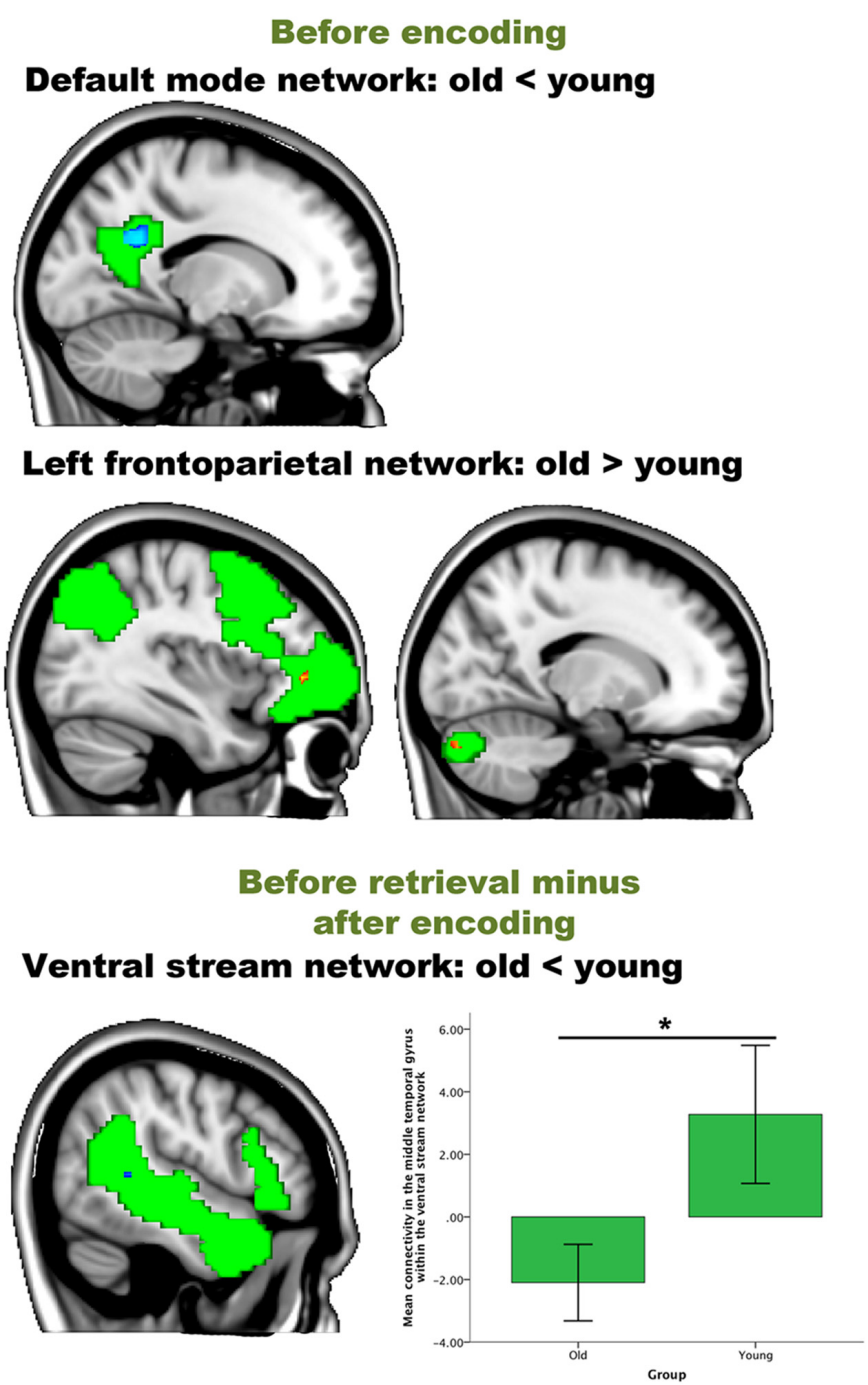
**Resting-state differences between older and young within the networks of interest**. Within-network group differences were found for the contrasts “before encoding” and “before retrieval minus after encoding.” Blue clusters show decreased connectivity in older adults, red clusters indicate increased connectivity in older adults. The bar chart depicts the group differences between the lateral temporal cortex and the ventral stream (error bars indicate 1 standard error) ^*^p-value < 0.05.

We then performed an interaction analysis “group by time point” for the three different time comparisons (see Figure 6). For the time point “before encoding,” significant group differences were again found in the default mode network and the left frontoparietal network. Older individuals showed less connectivity in the posterior cingulate cortex/precuneus of the default mode network [peak (*x, y, z* = −15, −54, 27), *p* = 0.003], compared to the younger participants. Older participants showed more connectivity in the frontal pole [peak (*x, y, z* = −36, 42, 0), *p* = 0.005] and [peak (*x, y, z* = −27, −54, −3), *p* = 0.016] and the crus I and II of the cerebellum of the frontoparietal network [peak (*x, y, z* = 15, −84, 30), *p* = 0.012] compared to their younger counterparts.

For the contrast “after encoding minus before encoding,” we found no significant group differences within any component (hypothesis 1). For the contrast “before retrieval minus after encoding” (reflecting hypothesis 2), we found less connectivity in the middle temporal gyrus of the ventral stream network [peak (*x, y, z* = 51, −42, 6), *p* = 0.039] for older participants than for younger participants (see Table 5 and Figure 6). No significant correlations were found between the within-network significant connectivity clusters and the retrieval performance on the experimental task (all *p* > 0.05; hypothesis 3).

**Table 5:** **Group differences in task-free connectivity within the networks of interest**.

**Brain regions within the clusters**	**Hemisphere**	**Cluster size (mm^3^)**	**Peak *p*-value**	**MNI coordinates (*x, y, z*)**
**BEFORE ENCODING**				
***The default mode network (old < young)***				
Posterior cingulate cortex, precuneus	LH	462	0.003	−15, −54, 27
***The left frontoparietal network (old > young)***				
Crus I and II	RH	72	0.012	5, −84, −30
Frontal pole, orbitofrontal cortex	LH	32	0.005	−36, 42, 0
Frontal pole	LH	12	0.016	−27, 54, −3
**BEFORE RETRIEVAL MINUS AFTER ENCODING**				
***The ventral stream network (old before retrieval – old after encoding > young before retrieval – young after encoding)***				
Middle temporal gyrus, posterior supramarginal gyrus	RH	16	0.039	51, −42, 6

*p-Values refer to the peak of all significant voxels. All p-values < 0.05 are family-wise error (FWE)-corrected at 0.05. Clusters with less than 10 mm^3^ are not reported. MNI, Montreal Neurological Institute; LH, left hemisphere; RH, right hemisphere. The Harvard-Oxford (sub)cortical and cerebellum atlas provided within FSL were used as anatomical reference*.

### Between network connectivity differences

The within-group repeated measures analyses for the coupling between the right frontoparietal network and the default mode network revealed for the older individuals [*F*_(2, 38)_ = 2.749, *p* > 0.05, η = 0.126] and for the younger participants [*F*_(2, 38)_ = 2.505, *p* > 0.05, η = 0.117] a non-significant effect of time. For the coupling between the executive network and the default mode network, we observed a significant effect of time in the young participants [*F*_(2, 38)_ = 8.139, *p* = 0.001, η = 0.300]; a significant *post-hoc* contrast was found between “after encoding and before retrieval” [*F*_(1, 19)_ = 16.986, *p* = 0.039, η = 0.205], but not in the older participants time [*F*_(2, 38)_ = 2.694, *p* > 0.05, η = 0.124].

**Figure 7:**
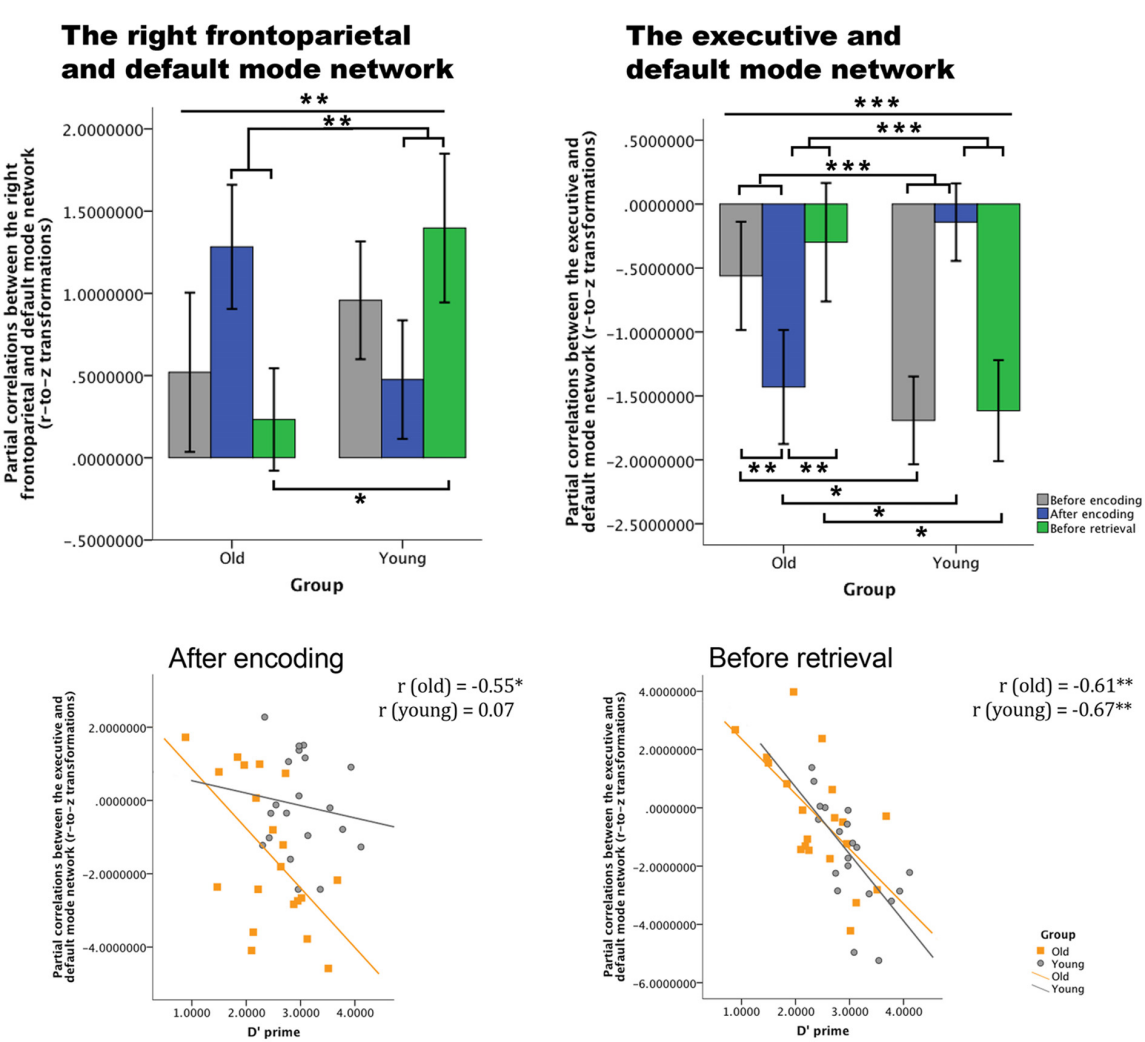
**Differences over time between young and old in across-network coupling and the association with memory performance**. **Top row:** partial correlations (*r*-to-*z* transformed) between networks over time for both groups (error bars indicate 1 standard error). **Bottom row:** correlations between on the one hand the partial correlations values of the coupling between the default mode network and the executive network, and on the other hand the *d*′ prime parameter for both groups. There is a significant association for older adults after encoding and before retrieval, for younger participants only before retrieval. ^*^p < 0.05; ^**^p < 0.01; ^***^p < 0.001.

Following our second hypothesis, we examined group differences in consolidation over time. The repeated measures ANOVA revealed a significant time by group interaction for the coupling between the right frontoparietal network and the default mode network [*F*_(2, 76)_ = 5.179; *p* = 0.008, η = 0.120], and between the executive network and the default mode network [*F*_(2, 76)_ = 9.428, *p* < 0.001, η = 0.199] (see Figure 7). As these interactions are disordinal, the main effects should not be interpreted, but are provided for completeness. For the coupling between the right frontoparietal network and the default mode network, the main effect of time was not significant [*F*_(2, 76)_ = 0.103; *p* > 0.05, η = 0.003] and the main effect of group is also not significant [*F*_(1, 38)_ = 0.382, *p* > 0.05, η = 0.010]. For the coupling between the executive network and the default mode network, we also found no significant main effects for time [*F*_(2, 76)_ = 0.517; *p* > 0.05, η = 0.013] or group [*F*_(1, 38)_ = 0.878; *p* > 0.05, η = 0.023]. In order to understand the direction of the effects, we have analyzed the simple effects. *Post-hoc* contrasts (type: repeated) showed that the interaction time by group for the coupling between the right frontoparietal network and the default network was based upon a significant difference between “after encoding” and “before retrieval” [*F*_(1, 38)_ = 10.844, *p* = 0.002]. This means that coupling “after encoding” was higher for older individuals than young ones, while the coupling “before retrieval” was higher for the young group compared to the older adults. There was no significant interaction between “before encoding” and “after encoding [*F*_(1, 38)_ = 3.60, *p* > 0.05]. Further exploration of the simple main effects showed a significant group difference for “before retrieval” (young > old) partial correlations between the networks [*F*_(1, 38)_ = 4.498, *p* = 0.041], but not for “before encoding” [*F*_(1, 38)_ = 0.529, *p* > 0.05] or “after encoding” [*F*_(1, 38)_ = 2.390, *p* > 0.05]. There were no within group differences for the paired time point comparisons. For the coupling between the executive and the default mode network, a significant time by group interaction was found between “before encoding” and “after encoding” [*F*_(1, 38)_ = 14.987, *p* < 0.001], and between “after encoding” and “before retrieval” [*F*_(1, 38)_ = 16.160, *p* < 0.001]. Figure 7 shows that there is indeed a higher competition (negative coupling) between these networks “before encoding” for the young group compared to the older group and “after encoding” the competition strength reverses (old > young). “Before retrieval” the competition is higher for the young group than for the older group. Further exploration of the simple main effects showed that the partial correlations between both networks differed significantly between older and young participants for the “before encoding” [*F*_(1, 38)_ = 4.306, *p* = 0.045], “after encoding” [*F*_(1, 38)_ = 5.719, *p* = 0.022], and “before retrieval” [*F*_(1, 38)_ = 4.679, *p* = 0.037] time points. Within the young group there were no significant differences in partial correlations between the paired time points. However, the older group showed significant differences between the “before encoding” and “after encoding” (*p* = 0.004), and the “after encoding” and “before retrieval” (*p* = 0.008).

To understand the functional relevance (hypothesis 3) of these changes in network couplings, we correlated these results with behavioral retrieval scores. Significant correlations between *d*′ prime values and the partial correlations between the executive and the default mode network were found for the older group after encoding (*p* = 0.018) and for both groups before retrieval (for old: *p* = 0.007; for young: *p* = 0.002) (see Figure 7).

## Discussion

The aim of this study was to investigate age-related changes during initial consolidation with a focus on large-scale functional networks. We expected age-related differences in initial consolidation by comparing connectivity patterns within relevant resting-state networks after encoding with those before encoding. Second, as consolidation is a dynamic process, we expected age-related differences over time. To that end, we set out to compare connectivity patterns of resting-state patterns of two different time frames after encoding.

Our results provided evidence that in older participants, compared to younger participants, dynamic changes in functional network coupling occur during the formation of new memory traces, i.e., the initial phase of consolidation. Our findings are consistent with animal lesion studies (Freeman and Gabriel, 1999) which suggested that mnemonic representations change over time and involve multiple functional networks. More importantly, however, our results extend these prior studies by revealing three novel findings: First, while we indeed observed an involvement of multiple, large-scale functional networks, it was the interaction between networks, and not changes within networks, that were related to age-associated differences during consolidation: increased competition between the default mode and executive network and increased cooperation between the default mode and frontoparietal network in the older group compared to the young group were detected during consolidation (Figure 7). Second, this coupling between networks showed a dynamic age-related reorganization over time, reflected in the non-static level of cooperation or competition between networks over time for both age groups (Figure 6). Third, the magnitude of alterations in coupling between networks was significantly associated with effective consolidation, especially in the older group. Thus, the dynamic organization of resting-state connectivity fluctuations amongst large-scale networks seems to be a powerful tool to measure the formation of new memory traces.

We first set out to investigate age-related differences in initial consolidation across large-scale functional networks. The time frame to stabilize or consolidate memories has always been expected to last weeks to years, but can be separated in an early and long-lasting, also termed synaptic and system consolidation. Synaptic consolidation is a fast process, occurring within the first minutes to hours after encoding. This type of consolidation is assumed to take place in the local nodes of the neuronal circuits that encode the internal representation of the event to be remembered (Dudai, 2004). In contrast, system consolidation, is supposed to take weeks, months or even years before it is achieved. This long-lasting consolidation is thought to induce network reorganization of areas involved in encoding (Dudai, 2004). It has long been thought that sleep is necessary to consolidate information by stimulating specific synaptic connections and removing redundant connections (Euston et al., 2007; Girardeau et al., 2009). Crucially, our results now suggest that new memory traces can indeed be formed in the awake resting-state state (Albert et al., 2009). Furthermore, such network modulations are detectable immediately after presentation of the material to be encoded (Figures 4, 6).

This is, to our knowledge, the first study on age-related neural changes during episodic memory consolidation. Consistent with predictions from Takashima et al. (2009), we found increased connectivity between the parahippocampal gyrus and the salience network in the older compared to the young group (Table 3), showing increased dependence of the medial temporal areas immediately after encoding. The salience network is known to be involved in bottom-up detection of salient events or processes, and mediates the switching between attention and memory-related networks (Bressler and Menon, 2010). Older adults seem to rely more on memory-related areas during early consolidation, compared to young adults in the post encoding phase. During the encoding of the items, young adults had higher activity in the parahippocampal gyrus compared to the older individuals (Supplemental Data). This switch in parahippocampal involvement might indicate that neocortical reorganization and independence from the medial temporal lobe areas occurs faster in younger individuals compared to the older group.

The second goal of this study was to investigate the temporal dynamics in age-related differences in consolidation across large-scale functional networks. When investigating changes in intrinsic functional connectivity between before retrieval vs. after encoding, we observed changes in the within-network analyses (Figure 6). Older individuals had lower connectivity values between the middle temporal gyrus and the ventral stream compared to young participants. This might provide additional evidence that consolidation in older individuals at that moment is still hippocampus-dependent. Younger individuals seem to recruit, potentially hippocampus-independent, functionally relevant networks, such as the ventral stream, early on in the consolidation process. The ventral stream is functionally important for form-representation (Kravitz et al., 2011). Our memory task involved memorizing objects and during encoding we did indeed observe activity in the inferior temporal and fusiform gyrus, areas part of the ventral stream (Supplemental Data). Since there was no correlation with retrieval and thus since these neural changes might not necessarily be related to consolidation, we remain cautious with these interpretations.

These findings are on par with prior studies, showing changes in BOLD signal fluctuations during rest indicative of off-line consolidation briefly after learning (Albert et al., 2009). Resting-state states have been shown to be suitable for investigating consolidation (Takashima et al., 2009; Tambini et al., 2010; Wang et al., 2012). However, findings remain controversial with respect to which networks might be involved in memory consolidation. The hippocampus seems to be a central hub for consolidation processes, but both enhanced as well as decreased connectivity to other brain regions has been reported (Takashima et al., 2009; Tambini et al., 2010; Vilberg and Davachi, 2013). Other areas, such as the fusiform gyrus or posterior parietal areas, are most likely related to the task at hand, and might reflect ongoing neocortical integration of the newly learned information. These apparent inconsistencies can be related to differences in analysis approaches (resting-state or task-based, region-of-interest based or network-based), population (only young individuals), or consolidation phase. Whether these neural changes have implications for later memory performance has also led to inconsistencies in the literature. While Tambini et al. (2010), Wang et al. (2012) and Vilberg and Davachi (2013) found correlations with behavioral performance in different brain areas, Takashima et al. (2009) found no correlation.

We found significant correlations with retrieval performance when assessing alterations in the coupling between networks, specifically between the executive and default mode network. The coupling between the executive and default mode network changed even over a short time period. After encoding, older participants showed increased anti-correlation compared to before encoding and to younger participants. As this correlated positively with retrieval accuracy, the most parsimonious explanation is that the effective formation of memory traces in older participants depends upon suppressing interference from competing networks (Kelly et al., 2008; Wermke et al., 2008). Just before retrieval, this competition is reduced, but is still important as indicated by the correlation with retrieval accuracy. This suggests that over a short period of time, first memory traces are laid while being protected from interference from other input. Younger participants are less vulnerable to such interference compared to older participants as indicated by their lower levels of anti-correlation after encoding. There were no significant pairwise changes over time in the young group, suggesting that the level of interference is not influencing consolidation effectiveness in young individuals. However, the significant interaction revealed that young individuals showed more anti-correlation before retrieval compared to after encoding than older individuals. As the before-retrieval network couplings correlated with retrieval performance in young participants, these fluctuations are consistent with existing consolidation theories, suggesting that the executive network becomes gradually independent from the default mode or memory-related network, and that effective memory traces are being formed.

Multiple memory processes cannot be attributed to one or a few brain regions (Buckner and Wheeler, 2001; Craik and Rose, 2012). It is therefore very likely that consolidation depends upon the dynamic interaction between large-scale networks. Our results do not allow a definite conclusion on whether or not these alterations in network coupling reflect a shift from a hippocampus-based network to a neocortical network or that both circuits remain toggling with each other, as long-term consolidation takes days or weeks. Taking these changes into account, it is likely, however, that protein synthesis alterations associated with long-term potentiation occur in this time frame (Reymann and Frey, 2007). Our data contributes to a growing body of literature by showing that consolidation at the system level, i.e., the coupling across networks, occurs already early on and is more strongly related to consolidation than within network changes. Reconsolidation, the susceptibility of memories to change by retrieving or reactivating them, has also been shown to be time dependent and network dependent (Alberini and Ledoux, 2013; Sandrini et al., 2013). However, the focus of this work was to investigate processes during consolidation and not during reconsolidation.

One might argue that participants were actively rehearsing the stimuli for later retrieval and that this effect is reflected in the BOLD signal changes. Actively rehearsing material supports consolidation. We cannot rule this out completely, but consider it unlikely that our participants adopted this strategy as we did not find activation patterns in the encoding phase that can be associated with internal mental repetition (e.g., Broca's areas) (D'esposito et al., 1997; Addis et al., 2007) and we believe that this task consists of too many items to be easily mentally rehearsed. Future studies may take this concern into account this by asking participants about their mental activities or by not informing participants about the goal of the task. Furthermore, we did not include a control session in which participants did neither perform a memory task nor another cognitive task. Importantly, prior work has shown that BOLD fluctuations during rest after learning are a good tool to investigate consolidation (Albert et al., 2009; Vincent, 2009; Daselaar et al., 2010). Another limitation of this study is related to the fact that functional connectivity differences during rest can be related to arousal fluctuations, such as fatigue. Although we did not collect heart rate measurements during scanning, after scanning we asked participants to rate their fatigue levels (1 = no fatigue, 5 = very fatigued) and whether they believed that they had fallen asleep during scanning. Both groups reported intermediate levels of fatigue [average (standard deviation)] for the three resting-state sessions for the older group: 1.7 (0.7), 2.1 (1.0), 2 (1.2); for the young group: 2.2 (1.2), 3.1 (1.3), 3.1 (1.4). One older subject indicated that she might have slept during the after-encoding and before-retrieval resting-state scan. Thus, even though we cannot rule out completely that fatigue may have influenced our data, its impact is most likely limited.

To conclude, we observed that age-related effective formation of new memory traces does not dependent on specific regions, or specific changes within networks, but rather depends upon the dynamic reorganization of competition and cooperation between resting-state large-scale networks over time. These findings are relevant for our understanding of the neural correlates of age-related differences in consolidation.

## Author contributions

Heidi I. L. Jacobs, Oezguer A. Onur and Juraj Kukolja were involved in the design of the study. Juraj Kukolja and Yasemin Göreci acquired to the data for this study. Heidi I. L. Jacobs, Kim N. H. Dillen and Okka Risius were responsible for the analyses of this study. Heidi I. L. Jacobs, Juraj Kukolja and Gereon R. Fink interpreted the results. All authors revised the work critically, approved the final version to be published and agreed to be accountable for the accuracy and integrity of the work.

### Conflict of interest statement

The authors declare that the research was conducted in the absence of any commercial or financial relationships that could be construed as a potential conflict of interest.
