# Supplementary material for: Consolidation in older adults depends upon competition between resting-state networks
Source: Front Aging Neurosci. 2015 Jan 9;6:344. doi: 10.3389/fnagi.2014.00344 (PMC4288239; doi:10.3389/fnagi.2014.00344)

***Supplementary Material***

**Consolidation in older adults depends upon competition between resting-state networks**

**Heidi IL Jacobs^1,*^, Kim N. Dillen^1^, Okka Risius^1^, Yasemin Göreci^2^, Oezguer A. Onur^1,2^, Gereon R. Fink^1,2^, Juraj Kukolja^1,2^**

^1^ Cognitive Neuroscience, Institute of Neuroscience and Medicine (INM3), Research Centre Jülich, Jülich, Germany;

^2^ Department of Neurology, University Hospital of Cologne, Cologne, Germany

*** Correspondence:** Dr. Heidi IL Jacobs, PhD, Cognitive Neuroscience, Institute of Neuroscience and Medicine (INM3), Research Centre Jülich, Leo-Brandt Str 5, 52425 Jülich, [h.jacobs@fz-juelich.de](mailto:h.jacobs@fz-juelich.de), Tel: +49 2461 61-4190; Fax:  +49 2461 61-1518

1. **Supplementary Methods**
   1. **Task-related fMRI analyses**

All preprocessing calculations and image manipulations were performed using Matlab (The MathWorks Inc., Natick, MA, USA) and SPM8 (statistical parametric mapping software, Wellcome Department of Imaging Neuroscience,London, UK; http://www.fil.ion.ucl.ac.uk). The images of each time series were spatially realigned to the first image to correct for head movements between scans and were synchronized to the middle slice to account for differences in slice acquisition time. The EPI images were then spatially normalised to the EPI template provided by SPM8 (resampled to 3mm×3mm×3mm voxels). Subsequently, EPI images were smoothed with a Gaussian kernel of 8mm (full-width-half-maximum) in order to meet the statistical requirements of the theory of Gaussian and to compensate for normal variation in individual brain anatomy across subjects.

During encoding, items which were correctly recognized as “old” in the subsequent retrieval session were divided into two subtypes:

- correct spatial context encoding (CorSCE): items associated with a correct spatial context judgement in the subsequent retrieval session;
- falsely spatial context encoding (FalSCE): items associated with a false spatial context judgement in the subsequent retrieval session

During retrieval, items were labelled analogously:

- correct spatial context retrieval (CorSCR): items associated with a correct spatial context judgement in the retrieval session;
- false spatial context retrieval (FalSCR): items associated with a false spatial context judgement in the retrieval session

The two sessions (encoding, retrieval) were analyzed separately.

We employed a random effects model. At the first level, the data of each subject were analyzed by defining four event types At the first level, the data of each subject were analyzed by defining four event types for the encoding session, consisting of three effects of interest (CorSCE, FalSCE, Subsequently forgotten (SF)) and one effect of no interest (missed responses in the encoding or retrieval sessions). For the retrieval session, five event types were defined: two effects of interest (CorSCR, FalSCR) and three effects of no interest (missed responses, New Correct, Old as New: the latter were of no interest since, during the retrieval run, subjects in these cases responded with their left instead of the right hand, making a comparison between activity underlying object recognition and non-recognition flawed by different laterality of response hands). The head movement parameters were included as additional regressors.

In an event-related fashion, each experimental condition was modeled using a boxcar reference vector convolved with a canonical hemodynamic response function and its first-order temporal derivative. The boxcar length for each event was determined by the corresponding reaction time in order to account for variable processing times. Low-frequency signal drifts were filtered using a cut-off period of 128 sec. Parameter estimates were subsequently calculated for each voxel using weighted least squares to provide maximum likelihood estimators ([Kiebel, 2003](#_ENREF_31)). No global scaling was applied. The parameter estimates for the HRF and linear contrasts of these estimates comprised the data for the second level analysis. At the second level we performed a flexible factorial ANOVA for the encoding session. The ANOVA comprised the within-subject factors subject, spatial, and item memory (CorSCE, FalSCE, SF), and the between-subject factor age group (young, older). F-Tests were performed for the main effect of spatial context judgment (CorSCE <> FalSCE). Furthermore, an F-Test was performed for the subsequent object memory effect [(CorSCE + FalSCE) <> SF]. Furthermore, interactions between age-group and spatial context memory as well as age group and item memory were tested.

The flexible factorial ANOVA of the retrieval session comprised the within- subject factors subject, spatial memory (CorSCR, FalSCR), and the between-subject factor age group (young, older). An F-Test were performed for the main effect of spatial context judgment (CorSCR <> FalSCR). Furthermore, interactions between age-group and spatial context memory were tested.

The tests resulted in an *F*-statistic for every voxel. The resulting set of voxel values for each contrast constituted a statistical parametric map of the *F*-statistic SPM_(F)_, which was subsequently transformed to the unit normal distribution SPM_(z)_.

Since these analyses were of an exploratory nature, activations are reported at p < 0.001, uncorrected for multiple comparisons.

The anatomical localization of significant activations and volume differences was assessed by reference to the MNI (Montreal Neurological Institute, Montreal, Canada) standard stereotactic space which approximates the Talairach system.

1. **Supplementary Results**
   1. **Task-related fMRI results**

During encoding, we found no main effect of spatial contextual memory but a significant main effect of item memory involving the middle frontal gyrus, fusiform gyrus, inferior and superior temporal gyrus, the parahippocampal gyrus, amygdala and cerebellum. There was no interaction between group and spatial contextual memory. We did find an interaction between group and item memory in the parahippocampal gyrus, where young individuals had higher activation levels than old (see Supplemental table 1).

During retrieval, we only found a main effect of spatial contextual memory in the medial and superior frontal gryus, the middle and superior temporal gyrus, the precental and postcentral gyrus, the insula, the hippocampus/parahippocampal gyrus, amygdala and cerebellum (see Supplemental table 2).

1. **Figures and Tables**

**Supplemental table 1: Activation effects for encoding**

| **Encoding** | | | | | | | | |
| --- | --- | --- | --- | --- | --- | --- | --- | --- |
| **Region** |  | **x** | **y** | **z** | **Z** | **p** | **voxels** | **effect** |
| **Main effect of spatial context memory (p<0.001, uncorrected)** | | | | | | | | |
| no significant effects |  |  |  |  |  |  |  |  |
|  |  |  |  |  |  |  |  |  |
| **Main effect of item memory (p<0.001, uncorrected)** | | | | | | | | |
| Middle Frontal Gyrus | R | 54 | 34 | 24 | 3.94 | <0.0001 | 49 | (CorSCE+FalSCE)>SF |
|  | L | -30 | 34 | -12 | 3.89 | <0.0001 | 71 | (CorSCE+FalSCE)>SF |
| Rectal Gyrus | R | 2 | 26 | -22 | 3.87 | <0.0001 | 46 | (CorSCE+FalSCE)>SF |
| Superior Temporal Gyrus | L | -42 | 8 | -20 | 3.65 | <0.0001 | 39 | (CorSCE+FalSCE)>SF |
| Amygdala | L | -26 | 0 | -12 | 3.27 | <0.0001 | 12 | (CorSCE+FalSCE)>SF |
| Fusiform Gyrus | L | -32 | -34 | -20 | 4.46 | <0.0001* | 202 | (CorSCE+FalSCE)>SF |
| Parahippocampal Gyrus | R | 28 | -36 | -16 | 3.80 | <0.0001 | 32 | (CorSCE+FalSCE)>SF |
| Inferior Temporal Gyrus | R | 50 | -54 | -10 | 4.87 | <0.0001 | 111 | (CorSCE+FalSCE)>SF |
|  | L | -44 | -56 | -14 | 3.79 | <0.0001 | 75 | (CorSCE+FalSCE)>SF |
|  | L | -38 | -62 | -2 | 4.33 | <0.0001 | 61 | (CorSCE+FalSCE)>SF |
| Cerebellum | R | 34 | -68 | -32 | 3.61 | <0.0001 | 27 | (CorSCE+FalSCE)>SF |
|  | L | -6 | -72 | -24 | 4.46 | <0.0001 | 88 | (CorSCE+FalSCE)>SF |
|  | R | 14 | -78 | -32 | 4.30 | <0.0001 | 90 | (CorSCE+FalSCE)>SF |
| Middle Occipital Gyrus | L | -36 | -80 | 16 | 3.68 | <0.0001 | 48 | (CorSCE+FalSCE)>SF |
|  |  |  |  |  |  |  |  |  |
| **Interaction between age group and spatial context memory (p<0.001, uncorrected)** | | | | | | | | |
| no significant effects |  |  |  |  |  |  |  |  |
|  |  |  |  |  |  |  |  |  |
| **Interaction between age group and item memory (p<0.001, uncorrected)** | | | | | | | | |
| Parahippocampal Gyrus | L | -26 | -42 | -2 | 3.83 | <0.0001 | 25 | young((CorSCE+FalSCE)>SF)>old((CorSCE+FalSCE)>SF) |
|  |  |  |  |  |  |  |  |  |

*P-values refer to the peak of all significant voxels. L = left hemisphere; R = right hemisphere. *: significant after FWE correction*

**Supplemental Figure 1: Main effect of item memory during encoding**

**P**

**L**

**z = -10**

**z = -54**

Note: Depicted is the inferior temporal gyrus

**Supplemental Figure 2: Interaction between age group and item memory during encoding**

**L**

**P**

z = -2

y = -42

Note: depicted is the left parahippocampal gyrus

**Supplemental Table 2: Activation effects for retrieval**

| **Retrieval** | | | | | | | | |
| --- | --- | --- | --- | --- | --- | --- | --- | --- |
| **Region** |  | **x** | **y** | **z** | **Z** | **p** | **voxels** | **effect** |
| **Main effect of spatial context memory (p<0.001, uncorrected)** | | | | | | | | |
| Superior Frontal Gyrus | L | -10 | 56 | 40 | 4.30 | <0.0001 | 88 | CorSCR>FalSCR |
| Medial Frontal Gyrus | L | -8 | 56 | 8 | 3.36 | <0.0001 | 34 | CorSCR>FalSCR |
| Superior Frontal Gyrus | L | -16 | 34 | 42 | 3.39 | <0.0001 | 16 | CorSCR>FalSCR |
| Basal Forebrain | R | 14 | 8 | -8 | 4.61 | <0.0001* | 378 | CorSCR>FalSCR |
| Superior Temporal Gyrus | L | -56 | 4 | -8 | 3.77 | <0.0001 | 16 | CorSCR>FalSCR |
| Insula | R | 46 | 2 | 6 | 3.82 | <0.0001 | 88 | CorSCR>FalSCR |
| Precentral Gyrus | L | -46 | -2 | 10 | 3.44 | <0.0001 | 10 | CorSCR>FalSCR |
| Amygdala | L | -24 | -4 | -12 | 4.55 | <0.0001* | 1134 | CorSCR>FalSCR |
| Middle Temporal Gyrus | R | 64 | -4 | -6 | 3.57 | <0.0001 | 13 | CorSCR>FalSCR |
| Middle Temporal Gyrus | L | -54 | -20 | -10 | 3.75 | <0.0001 | 79 | CorSCR>FalSCR |
| Insula | L | -26 | -20 | 14 | 3.30 | <0.0001 | 12 | CorSCR>FalSCR |
| Paracentral Lobule | L | -4 | -22 | 50 | 3.70 | <0.0001 | 397 | CorSCR>FalSCR |
| Postcentral Gyrus | L | -50 | -24 | 50 | 3.64 | <0.0001 | 47 | CorSCR>FalSCR |
| Inferior Parietal Lobule | L | -42 | -28 | 24 | 3.91 | <0.0001 | 125 | CorSCR>FalSCR |
| Hippocampus/ Parahippocampal Gyrus | L | -40 | -34 | -14 | 4.91 | <0.0001* | 1489 | CorSCR>FalSCR |
| Cerebellum | R | 30 | -40 | -30 | 4.15 | <0.0001* | 600 | CorSCR>FalSCR |
| Insula | L | -48 | -40 | 20 | 3.49 | <0.0001 | 11 | CorSCR>FalSCR |
| Parahippocampal Gyrus | L | -34 | -46 | -6 | 3.35 | <0.0001 | 13 | CorSCR>FalSCR |
| Cingulate Gyrus | L | -16 | -50 | 26 | 4.34 | <0.0001 | 99 | CorSCR>FalSCR |
|  |  |  |  |  |  |  |  |  |
| **Interaction between age group and spatial context memory (p<0.001, uncorrected)** | | | | | | | | |
| no significant effects |  |  |  |  |  |  |  |  |
|  |  |  |  |  |  |  |  |  |

*P-values refer to the peak of all significant voxels. L = left hemisphere; R = right hemisphere. *: significant after FWE correction*

**Supplemental Figure 3: Main effect of spatial contextual memory during retrieval**

**z = -14**

**y = -34**

**P**

**L**

Note: depicted is a cluster involving the hippocampus and parahippocampal gyrus

**Supplemental Figure 4: Overview of the processing steps**


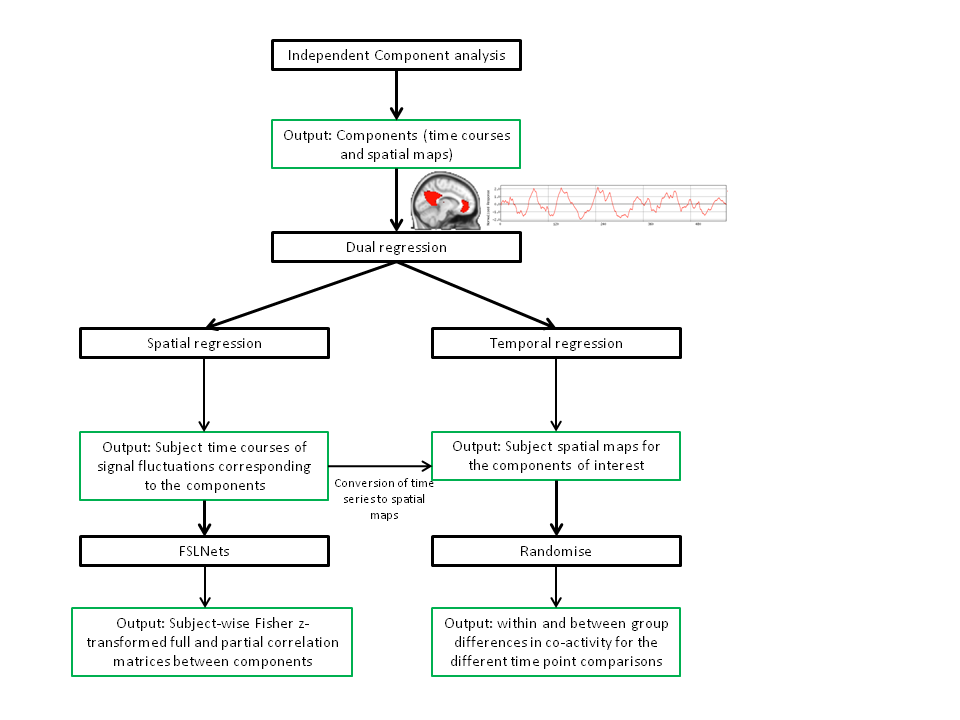


**Supplemental Figure 5: Within-group effects for the before encoding time period within the whole brain**


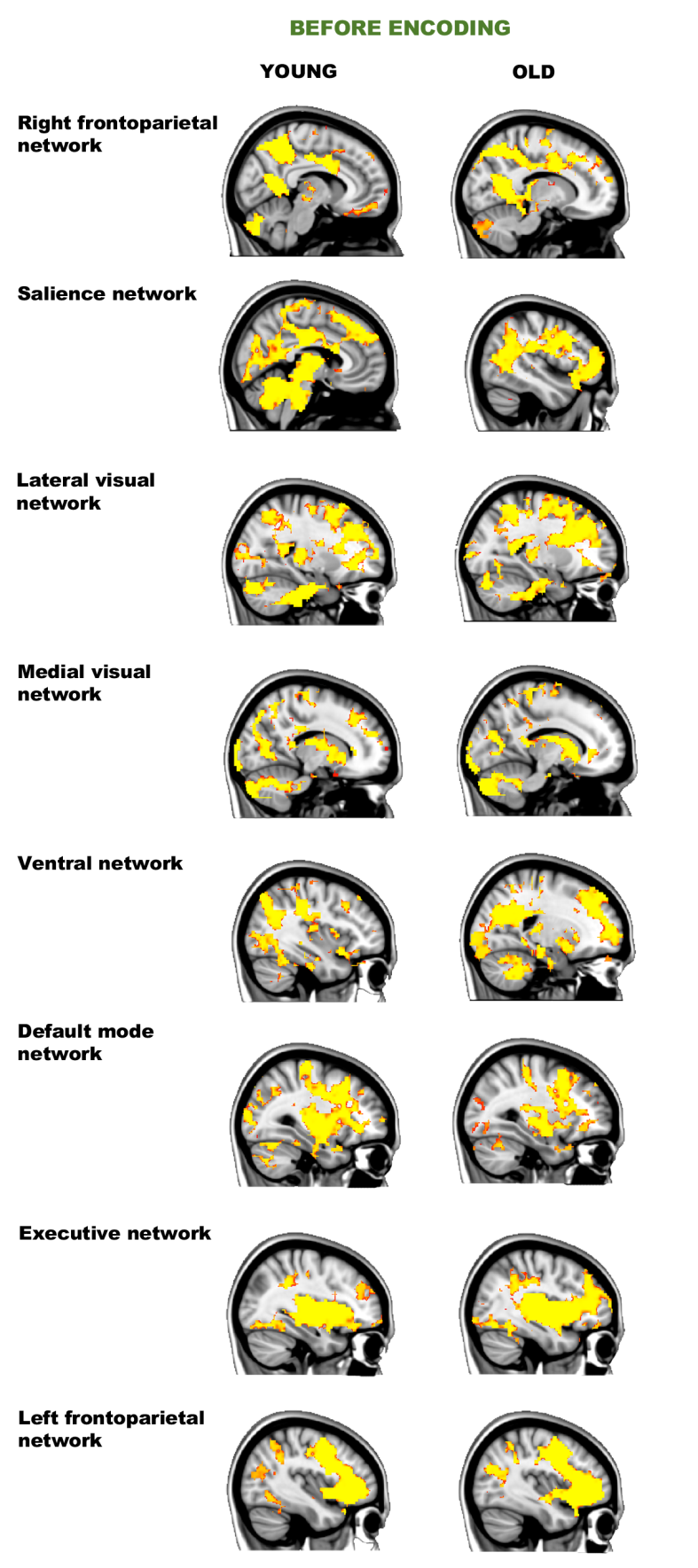


**Supplemental Figure 6: Within-group effects for the before encoding time period within the resting-state networks of interest**


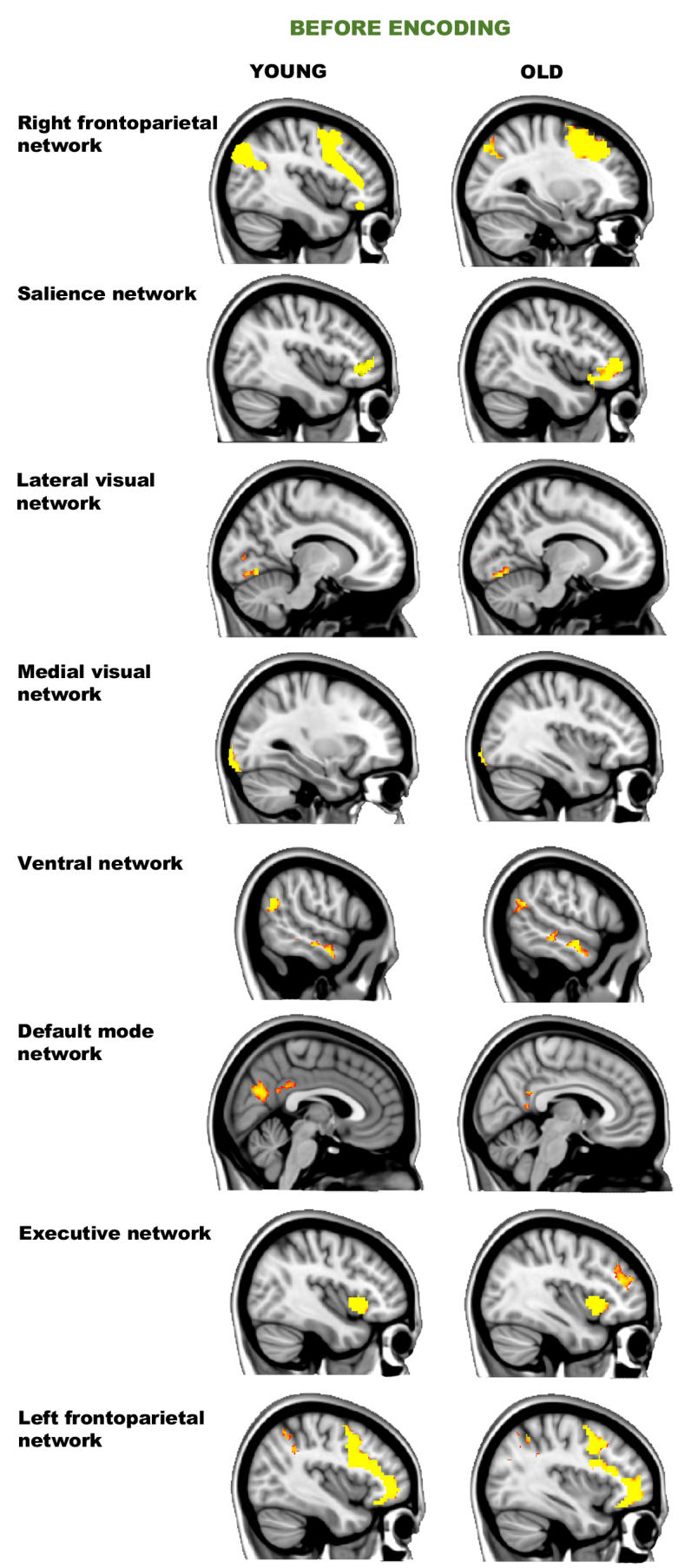

Supplement: Supplementary file 1 [file DataSheet1.DOCX]
